# Myosin phosphatase and RhoA-activated kinase modulate neurotransmitter release by regulating SNAP-25 of SNARE complex

**DOI:** 10.1371/journal.pone.0177046

**Published:** 2017-05-09

**Authors:** Dániel Horváth, István Tamás, Adrienn Sipos, Zsuzsanna Darula, Bálint Bécsi, Dénes Nagy, Judit Iván, Ferenc Erdődi, Beáta Lontay

**Affiliations:** 1 Department of Medical Chemistry, Faculty of Medicine, University of Debrecen, Debrecen, Hungary; 2 Hungarian Academy of Sciences, Proteomics Research Group, Biological Research Centre, Szeged, Hungary; 3 MTA-DE Cell Biology and Signaling Research Group, Faculty of Medicine, University of Debrecen, Debrecen, Hungary; Chinese University of Hong Kong, HONG KONG

## Abstract

Reversible phosphorylation of neuronal proteins plays an important role in the regulation of neurotransmitter release. Myosin phosphatase holoenzyme (MP) consists of a protein phosphatase-1 (PP1) catalytic subunit (PP1c) and a regulatory subunit, termed myosin phosphatase targeting subunit (MYPT1). The primary function of MP is to regulate the phosphorylation level of contractile proteins; however, recent studies have shown that MP is localized to neurons, and is also involved in the mediation of neuronal processes. Our goal was to investigate the effect of RhoA-activated kinase (ROK) and MP on the phosphorylation of one potential neuronal substrate, the synaptosomal-associated protein of 25 kDa (SNAP-25). SNAP-25 is a member of the SNARE (soluble N-ethylmaleimide sensitive factor attachment protein receptor) complex, along with synaptobrevin and syntaxin, and the primary role of SNAP25 is to mediate vesicle fusion. We showed that MYPT1 interacts with SNAP-25, as revealed by immunoprecipitation and surface plasmon resonance based binding studies. Mass spectrometry analysis and *in vitro* phosphorylation/dephosphorylation assays demonstrated that ROK phosphorylates, while MP dephosphorylates, SNAP-25 at Thr138. Silencing MYPT1 in B50 neuroblastoma cells increased phosphorylation of SNAP-25 at Thr138. Inhibition of PP1 with tautomycetin increased, whereas inhibition of ROK by H1152, decreased the phosphorylation of SNAP-25 at Thr138 in B50 cells, in cortical synaptosomes, and in brain slices. In response to the transduction of the MP inhibitor, kinase-enhanced PP1 inhibitor (KEPI), into synaptosomes, an increase in phosphorylation of SNAP-25 and a decrease in the extent of neurotransmitter release were detected. The interaction between SNAP-25 and syntaxin increased with decreasing phosphorylation of SNAP-25 at Thr138, upon inhibition of ROK. Our data suggest that ROK/MP play a crucial role in vesicle trafficking, fusion, and neurotransmitter release by oppositely regulating the phosphorylation of SNAP-25 at Thr138.

## Introduction

Exocytosis is an essential component of cell signaling throughout the body and underpins a diverse array of essential physiological pathways, even though exocytosis varies considerably between cell types and can require adaptations [1]. Neurotransmitter release is a specialized mechanism of exocytosis, which includes Ca^2+-^dependent release of neurotransmitters from synaptic vesicles [2]. The elevated calcium level is the key regulator of the process, but other regulatory elements have also been identified. In a nerve terminal, synaptic vesicle docking and release are restricted to an active zone. A pool of already docked vesicles resides at the presynaptic target membrane called the readily releasable pool of vesicles. A single calcium spike results in only a partial release of this pool, suggesting an additional level of regulation of neurotransmission [3]. The recycling pool contains 5–20% of all vesicles and is refilled continuously by newly synthetized vesicles depending on the physiological frequency of stimulation [4]. However, the majority of vesicles belong to the third pool type, the reserve pool, which provides a depot of synaptic vesicles from which release is triggered by intense stimulation [5].

The SNARE (soluble N-ethylmaleimide sensitive factor attachment protein receptor) complex is one of the highly conserved targets of regulated exocytosis. SNAREs are members of a family of proteins which form a complex and regulate neuronal exocytosis. The t-SNARES, such as syntaxin and synaptosomal-associated protein of 25 KDa (SNAP-25), are attached to the target membrane of the vesicles. Other components, such as synaptobrevin (VAMP), are located on the vesicle membrane (v-SNARES) and binds to its cognate t-SNARE [6, 7]. SNARE is believed to form a highly stable trimeric exocytotic complex [8] that generates a twisted bundle of four parallel α helices to bring the two membranes into close proximity and allow fusion [9]. The real number of releasable vesicles are modulated by the rate of priming and depriming of vesicles, and is related to the preassembling or the dissociation of SNARE complex, respectively [10].

Regulation of the SNARE complex, which is dependent on protein phosphorylation at serine/threonine (Ser/Thr) residues, is necessary for proper neuronal functions [11]. SNARE activation takes place in a step-wise fashion to enhance or to block its interactions. T-SNAREs must join and be delivered to the site of action before creating a complex with v-SNARE. Synaptotagmin is responsible for preventing vesicles from fusing to the membrane, even after they are docked, by interacting with SNAREs [7]. Phosphorylation of SNAREs by protein kinases also prevents the formation of the SNARE complex, when SNAREs are targeted to their site of action [12]. SNAP-25, which has multiple phosphorylation sites, is a major regulatory target of protein kinases and phosphatases [13]. SNAP-25 was found to be differentially phosphorylated by protein kinase A (PKA) and C (PKC) in neuroendocrine PC12 cell. PKC regulates refilling of the vesicle pools and recycling of SNAP-25. After the readily releasable pool has been depleted, SNAP25 phosphorylation by PKC makes elements of the complex more available to form SNARE complexes and dock more vesicles [14]. PKA phosphorylates the Thr138 residue of SNAP-25 specifically [15], and regulates the size of the readily releasable pool of vesicles, supposedly by modulating the protein binding properties of SNAP-25 [14].

Compared to the phosphorylation by protein kinases, dephosphorylation mechanisms catalyzed by protein phosphatases are poorly understood. The effects of phosphatase inhibitors on neuronal exocytosis have been investigated. Tautomycetin, a PP1-specific inhibitor, decreases depolarization-induced exocytosis, while Y27632, a ROK-specific inhibitor, has the opposite effect [16]. Myosin phosphatase (MP) first identified in smooth muscles, is a Ser/Thr specific protein phosphatase 1 (PP1) consisting of a 38 kDa catalytic subunit (PP1c) of the β/δ isoform, a 130/133 kDa myosin phosphatase target subunit-1 (MYPT1) and a 20 kDa subunit of largely unknown function [17]. MP (also termed PP1M) is involved in exocytotic processes; MYPT1 interacts and affects the dephosphorylation of several neuronal proteins [16]. Phosphorylation of Thr696 and/or Thr853 in MYPT1 by ROK results in the inhibition of MP [18, 19], and these regulatory events have also been identified in cortical synaptosomes [16]. MP is present in cortical synaptosomes in association with ROK [20], suggesting that MP is involved in synaptic signaling. ROK and MP regulate neurotransmission via phosphorylation/dephosphorylation of several presynaptic proteins (e.g., myosin-II, synapsin-I, syntaxin-1) that are involved in neurotransmitter release [16].

In the present study we tested the hypothesis that ROK and MP regulate SNAP-25 through reversible phosphorylation. Our results suggest that SNAP25 interacts with MP via binding to MYPT1. ROK phosphorylates SNAP-25 at the Thr138 and the dephosphorylation is catalyzed by MP. Phosphorylation of SNAP-25 by ROK results in a decrease in KCl-induced synaptosomal exocytosis, while dephosphorylation by MP accelerates the process. Our data suggest that ROK/MP enzymes play a crucial role in vesicle trafficking, fusion and neurotransmitter release through the regulation of the assembly of the SNARE complex.

## Materials and methods

### Materials

All chemicals were obtained from Sigma-Aldrich (St. Louis, MO, USA) unless otherwise indicated. Microcystin-LR was produced and purified as described previously [21].

### Antibodies

Anti-SNAP-25, anti-actin, anti-Flag, anti-GST, anti-syntaxin, peroxidase conjugated anti-rabbit IgG and anti-chicken IgG were purchased from Sigma-Aldrich (St. Louis, MO, USA). Anti-SNAP-25^pThr138^ (Abgent, San Diego, CA, USA), anti-MYPT1^1-296^ [20], anti-PP1cδ (Millipore, Billerica, MA, USA), anti-GAPDH (Santa Cruz, CA, USA), anti-CPI17^pThr38^ [22], horseradish peroxidase-linked anti-mouse IgG (Cell Signalling, Danvers, MA, USA), Clean-Blot IP Detection Reagent (Thermo Scientific, Waltham, MA, USA) and Texas Red-X phalloidin (Life Technologies, Carlsbad, CA, USA) were purchased as indicated. Alexa Fluor 488-conjugated anti-rabbit IgG, Alexa Fluor 546-conjugated anti-mouse IgG, Alexa Fluor 546-conjugated anti-goat IgG and To-Pro-3 iodide were obtained from Molecular Probes (Eugene, OR, USA).

### Western blotting

Solubilized proteins from cells or synaptosomes were boiled with 5x SDS-PAGE (sodium dodecyl sulphate-polyacrylamide gel electrophoresis) sample buffer (50% glycerol, 10% SDS, 0.31 M Tris, 100 mM dithiothreitol, 0.01% bromophenol blue) at 100°C for 5 min and proteins were separated by SDS-PAGE on 12% (w/V) polyacrylamide gel [23]. After gel electrophoresis, proteins were transferred to nitrocellulose membrane (Bio-Rad, Hemel Hempstead, UK). Membranes were incubated with blocking solution (5% bovine serum albumin (BSA) in Tris buffered saline (TBS: 136 mM NaCl, 2.7 mM KCl, 25 mM Tris-HCl pH 7.4) containing 0.1% Tween-20 (TBST) for 1 h at room temperature, and then with the primary antibodies overnight at 4°C. Membranes were washed with TBST and incubated with the secondary antibodies for 1–2 h at room temperature. Primary and secondary antibodies were diluted in TBST containing 0.5% BSA. Immunoreactions were developed by enhanced chemiluminescence and visualized using FluorChem FC2 Imager (Alpha Innotech, San Leandro, CA, USA). Densitometry analysis of immunoreactive bands was carried out using Image J software (.NIH Bethesda, USA).

### Immunoprecipitation

Anti-SNAP-25, anti-MYPT1^1-296^ and anti-PP1cδ antibodies were bound to protein-A Sepharose (PAS) resin (GE Healthcare, Little Chalfont, UK) in the presence of immunoprecipitation (IP) buffer (50 mM Tris-HCl pH 7.4, 150 mM NaCl, 1 mM EDTA, 1% Triton X-100) by incubation for 1 h at 4°C. Precleared (previously incubated with PAS) B50 neuroblastoma cell lysate was added to PAS-antibody complexes and incubated for 90 min at 4°C. After centrifugation (4°C, 900 g, 3 min) the supernatant was removed, and PAS-antibody-proteins complexes were washed with IP buffer twice, then once with phosphate buffered saline (PBS). The supernatant was removed and PAS-antibody-protein complexes were boiled at 100°C for 5 min with SDS-PAGE sample buffer, then the samples were subjected to Western blot analysis.

### Surface plasmon resonance

Interactions of MYPT1 and its mutants with SNAP-25 were analysed by surface plasmon resonance (SPR) using a Biacore 3000 instrument (GE Healthcare, Little Chalfont, UK). Anti-GST was coupled on the surface of a CM5 chip by amine-coupling, then full-length GST-MYPT1^WT^ or its C-terminal fragment (GST-MYPT1^667–1004^) was captured on the surface. His-tagged MYPT1 proteins (His-MYPT1^1–296^ or His-MYPT1^1–633^) were immobilized directly on CM5 sensor chips by amine-coupling. Determination of the binding of Flag-SNAP-25 on these surfaces was performed as described previously [24]. Kinetic parameters and the dissociation constant (*K*_D_) values were extracted from the sensorgrams with BIAevaluation 3.1 software using a 1:1 interaction model.

### *In vitro* kinase assay

Phosphorylation of purified Flag-SNAP-25 by RhoA-activated protein kinase (ROK) was performed in an *in vitro* kinase assay using radioactive phosphoryl donor substrate. Flag-SNAP-25 (5 μM) was incubated with or without (control) 20 ng/μl ROK (Millipore, Billerica, MA, USA) in the presence of 1 mM γ-[^32^P]ATP (Institute of Isotopes Co., Budapest, Hungary) and 1 μM microcystin-LR (MC-LR, a protein phosphatase inhibitor) for 120 min at 30°C in a ROK assay buffer containing 25 mM β-glycerophosphate, 0.5 mM EGTA, 0.5 mM dithiothreitol (DTT), 5 mM MgCl_2_, and 20 mM MOPS (pH 7.2). The reactions were terminated by the addition of hot SDS-PAGE sample buffer. After SDS-PAGE, the phosphorylation of proteins was verified by autoradiography of the gels.

### Protein phosphorylation/dephosphorylation assay

The lysate of tsA201 cells expressing Flag-SNAP-25 was incubated with Anti-Flag M2 affinity gel to pull down Flag-SNAP-25. Beads were washed with TBS, and incubated with ROK (final concentration: 20 ng/μl) and ATP (final concentration: 0.5 mM) for 30 min at 30°C in the presence 1 μM of microcystin-LR in ROK assay buffer. Control samples were handled in the same way without addition of ROK. After phosphorylation, the beads were washed twice with TBS and incubated in the presence or absence of the subunits of myosin phosphatase (purified Flag-MYPT1 and recombinant PP1cδ at a final concentration of 25 nM and 5 nM, respectively) for 15 min at 30°C. After washing with TBS, beads were incubated with SDS-PAGE sample buffer at 100°C for 5 min. Phosphorylation and dephosphorylation of SNAP-25 were analysed by Western blot using SNAP-25^pThr138^ phospho-specific antibody and anti-SNAP-25 for a loading control.

### Mass spectrometry analysis

Bands of interest were subjected to in-gel digestion using side-chain protected porcine trypsin (37°C, 4h). Approximately 80% of the peptide mixtures were subjected to phosphopeptide-enrichment using TiO_2_ [25]. The phosphopeptide fractions, as well as the remaining 20% of the original samples, were analyzed by data-dependent LC-MS/MS using an Orbitrap Elite mass spectrometer (MS spectra acquired in the Orbitrap, MS/MS spectra in the linear ion trap). Peak lists generated from the MS/MS data by the PAVA software [26] were searched against the human subset of the Swissprot database (downloaded 06/10/2014; 20265 target sequences concatenated with a randomized sequence for each entry) using the ProteinProspector search engine (v.5.10.9.). Search parameters: enzyme: trypsin with maximum 1 missed cleavage; fixed modification: carbamidomethyl (Cys); variable modifications: acetylation (protein N-terminus), oxidation (Met), pyroglutamic acid formation (N-terminal Gln) allowing 2 variable modifications per peptide; mass accuracy: 5 ppm and 0.8 Da for precursor and fragment ions (both monoisotopic), respectively. Subsequently another search was conducted on the subset of confidently identified proteins using the same search parameters except that 2 missed cleavages were allowed, and phosphorylation on Ser/Thr was added as variable modification allowing 3 variable modifications/peptide. For all searches the following acceptance criteria were applied: score>22 and 15, and E-value<0.01 and 0.05 for protein and peptide identifications, respectively. For phosphopeptide site assignments, SLIP score threshold [27] was set to 6 (indicating 95% confidence in site localization). All phosphopeptide assignments were inspected manually.

### Site-directed mutagenesis

Bacterial expression vector pReceiver-M13, which encodes wild type SNAP-25, was purchased from GeneCopoeia (Rockville, MD, USA). Point mutation of Thr138 of SNAP-25 to alanine was performed using QuickChange II XL Site-Directed Mutagenesis Kit (Qiagen, Hilden, Germany) and primers described in S1 Table. Newly synthetized mutant DNA was transformed into XL10-Blue supercompetent cells (Agilent, Santa Clara, CA, USA) and mutant plasmids were purified using EZ-10 Spin Column Plasmid DNA Minipreps Kit (Bio Basic, Markham, Canada). The sequence of the mutant was analysed and verified by DNA sequencing.

### Protein production and purification

Flag-SNAP-25 or Flag-KEPI plasmids (GeneCopoeia, Rockville, MD, USA) were transfected into tsA201 cells using polyethylenimine (PEI) transfection reagent. The plasmids (15 μg) and PEI (30 μl) were diluted in 150 mM sterile NaCl solution and incubated for 30 minutes. One ml transfection mixture was added to 4 ml serum-free Dulbecco’s modified Eagle’s medium (DMEM) and pipetted onto the adherent tsA201 cells (50–60% confluency). Six hours later 5 ml of 20% fetal bovine serum (FBS) containing DMEM was added to the cells, which were grown for an additional 18 hours before collecting for analysis

### Cell culture and treatment

B50 neuroblastoma cells (purchased from Sigma, St. Louis, MO, USA) were maintained in Dulbecco’s modified Eagle’s medium supplemented with 10% (V/V) FBS and 2 mM L-glutamine in a 5% CO_2_-humidified atmosphere at 37°C. Cells were plated into 6-well culture plates, the medium was removed after reaching a confluent state, and cells were synchronized via incubation in serum-free media for 24 h. To inhibit PP1 and ROK activity, tautomycetin (TMC) and H1152 (Santa Cruz Biotechnology, Santa Cruz, CA, USA) were added to the medium at concentrations of 5 μM and 10 μM for 1 h and 30 min, respectively. Cells were harvested in radioimmunoprecipitation assay (RIPA) buffer (25 mM Tris-HCl pH 7.6, 150 mM NaCl, 0.25% sodium deoxycholate, 1% NP-40, 0.1% sodium dodecyl sulphate) containing protease inhibitors (Roche, Basel, Switzerland) and 1 μM of microcystin-LR. Proteins were solubilized by sonication and protein concentrations were determined with Pierce BCA Protein Assay Kit (Thermo Scientific, Waltham, MA, USA).

### Gene silencing

The siRNA delivery was carried out with a reverse transfection protocol. Cells at 80% confluency were washed with sterile 1x PBS, trypsinized, and transfected with 100 nM double stranded siRNA (Thermo Scientific Inc., see S2 Table) consisting of a pool of 4 different siRNAs (S3 Table) targeting the endogenous MYPT1 subunit of MP. For the transfections, DharmaFECT 2 (Dharmacon, Lafayette, CO, USA) transfection reagent was used according to the manufacturer’s instructions. After 48 hours, cells were either subjected to MTT or phosphatase activity assays, or lysed and prepared for Western blot analysis. Gene knock down experiments were carried out in 6-well plates (1x10^5^ cells/well) for Western-blot and phosphatase activity assays and in a 96-well format for MTT assays (~3x10^3^ cells/well).

### Assay of protein phosphatase activity

B50 neuroblastoma cells were treated with TMC (5 μM) or H1152 (10 μM) inhibitors for 1 hour and 30 mins respectively and then lysed. Protein phosphatase activity in the lysates were measured using ^32^P-labelled 20 kDa myosin light chain (^32^P-MLC20) substrate in TM buffer (20 mM Tris-HCl, 0.1% β-mercaptoethanol, pH 7.4). The reaction was initiated by the addition of ^32^P-MLC20 at a final concentration of 1 μM. After 5 min of incubation at 30°C the reaction was terminated by the addition of 200 μl 10% trichloroacetic acid (TCA) and 200 μl 6 mg/ml BSA. After centrifugation, the ^32^P_i_ content of the supernatant was measured in a Tri-Carb 2800TR scintillation counter (PerkinElmer, Waltham, MA, USA).

### MTT assay

Viability of B50 cells were studied by MTT assay as described before [28]. Shortly, cells were synchronized in serum-free DMEM medium and were treated with TMC and H1152 as detailed before. Ten μl of MTT (3-(4,5-dimethylthiazol-2-yl)-2,5-diphenyltetrazolium bromide dissolved in PBS) solution (5 mg/ml) was added and the cells were incubated in a 5% CO_2_-humidified atmosphere at 37°C for 1 hour. The formazan crystals were dissolved in dimethyl sulfoxide (DMSO) and the absorbance was determined by a photometer (LabSystems Multiskan MS, Labsystems Diagnostics Oy, Vantaa, Finland) at 540 nm wavelength.

### Synaptosome preparation and treatment

Synaptosomes were prepared from C3H mouse cerebral cortex using an established protocol [16] with modifications detailed here. Mice (3-5/experiment) were euthanized by carbon dioxide inhalation. After decapitation, the cortex was removed and immersed in homogenization buffer (0.32 M sucrose, 1.0 mM EDTA, 0.25 mM DTT, pH 7.4) in a ratio of 10 ml ice-cold buffer to 1 g of cortex. Tissue was homogenized in a glass potter and the homogenate was centrifuged at 1000 g for 10 min at 4°C using an Allegra X-12R centrifuge (Beckman Coulter, Brea, CA, USA). The supernatant was collected into a new tube, the volume was increased to 12 ml with homogenization buffer, and two 2 ml volumes of the diluted supernatant were added slowly to the top of a Percoll gradient (2–2 ml of 23; 15; 10; 3% Percoll/homogenization buffer) in polycarbonate tubes. Tubes were centrifuged at 32500 g for 5 min at 4°C using Beckman L7-55 ultracentrifuge (Beckman Instruments Inc., Fullerton, CA, USA). Fractions between 10/15 and 15/23% phases were collected, diluted with four-times volume of Krebs buffer (118 mM NaCl, 5 mM KCl, 25 mM NaHCO_3_, 1 mM MgCl_2_, 10 mM D-glucose, pH 7.4), and centrifuged at 12600 g for 25 min at 4°C. The supernatant was removed and the pellet was resuspended in Krebs buffer and centrifuged again at 12600 g for 20 min at 4°C. The synaptosome pellet was resuspended in 3 ml of Krebs buffer and CaCl_2_ was added at a final concentration of 1.2 mM, then were incubated at 30°C for 1 h in the presence or absence of inhibitors (5 μM TMC or 10 μM H1152) and KCl.

The protocol for animal handling (5/2015/DEMÁB; 6/2011/DEMÁB) was authorized by the Animal Care and Protection Committee of the University of Debrecen, and was in accordance with the guidelines of the European Union Council, as well as the Hungarian regulations on the use of laboratory animals. Mice were given *ad libitum* access to food and water and were kept at 25°C temperature, applying a 12 hour light/12 hour dark cycle. The veterinarian at the University of Debrecen examined the health status of the mice every month, and in case of potential sickness, microbiological examinations are conducted and the appropriate interventions (therapy, quarantine) are applied. Mice were raised in the Experimental Animal House of the University of Debrecen, Debrecen, Hungary (XV-KÁT/2000) and supplied by Dr. Balázs Pál (licence number: 6/2011/DEMÁB). The Animal House is undergoing regular authority supervision every 2 years.

### Protein transduction into synaptosomes and determination of exocytosis

Purified Flag-KEPI protein [29] was introduced into synaptosomes according to a previously described protocol [30] with slight modification. Flag-peptide or Flag-KEPI (0.3 μg/μl) was added to the synaptosomes resuspended in HBS buffer (142 mM NaCl, 2.4 mM KCl, 1.2 mM K_2_HPO_4_, 1 mM MgCl_2_, 5 mM D-glucose, 0.1 mM EGTA, 10 mM HEPES) containing 25% (V/V) DMSO. No proteins or peptides were added to control samples. After freezing and thawing, samples were centrifuged at 4000 *g* for 3 min at 4°C and pellets were washed twice with Krebs buffer. Synaptosomes were resuspended in Krebs buffer at 1 mg protein/ml. After addition of 1.2 mM CaCl_2_ and 30 mM KCl, samples were incubated at 30°C for 30 min, then a Western blot or exocytosis assay was performed. The exocytosis assay was carried out as previously described [16]. Briefly, a water-soluble styryl dye, FM 2–10, is reversibly inserted into the outer leaflet of the cell membrane and becomes fluorescent. By endocytosis, the dye enters into the synaptosome. During subsequent exocytosis, the dye is released into the medium, so the intensity of the fluorescence decreases with neurotransmitter release ([1], [31]. Exocytosis was induced by 30 mM KCl using an established protocol according to Cousin and Robinson [32] which was modified for high-throughput as published by Lontay *et al* [16]. Synaptosomes suspended in Krebs buffer were incubated at 37°C for 3 minutes, then FM 2–10 was added. After a 1 minute incubation at 37°C, dye uptake was evoked by addition of 30 mM KCl. After incubating for 2 min at 37°C, synaptosomes were washed twice with Krebs buffer, centrifuged at 1000 *g* for 1 min, and the pellet was resuspended in Krebs buffer containing 1.2 mM CaCl_2_ and added to 96-well black plates. Exocytosis and neurotransmitter release induced by 30 mM KCl and the decrease of fluorescence was measured by Fluoroskan FL (excitation: 488 nm, emission: 540 nm).

### Preparing brain slices and treatment

Experiments were performed in artificial cerebrospinal fluid (aCSF) of the following composition: 120 mM NaCl, 2.5 mM KCl, 26 mM NaHCO_3_, 10 mM glucose, 1.25 mM NaH_2_PO_4_, 2 mM CaCl_2_, 1 mM MgCl_2_, 3 mM myo-inositol, 0.5 mM ascorbic acid, and 2 mM sodium pyruvate, pH 7.2. For the slice preparation, a modified aCSF (low sodium aCSF) was used where 95 mM NaCl was replaced by 130 mM sucrose and 60 mM glycerol. C3H mice (8 to 30 days old) of both sexes were used. After decapitation of the animal and removal of the brain, 200 μm thick coronal brain slices were prepared in ice-cold low sodium aCSF using a Microm HM 650 V vibratome (Microm International GmbH, Walldorf, Germany). After preparation, the slices were transferred into a Millicell CM culture plate insert (PICM01250; Millipore, Billerica, MA, USA) filled with aCSF. The culture plate inserts were placed in an incubating chamber which was designed for fluorescent dye loading [33]. Following the transfer, the incubating solution was slowly replaced with aCSF containing 10 μM H1152 or 5 μM tautomycetin with or without 8 mM KCl. The slices incubated for 60 min at room temperature under moderately pressurised carbogen (95% O_2_ 5% CO_2_) conditions. After incubation, the slices were either frozen in liquid nitrogen for Western blot analysis or mounted in Mounting Medium for cryotomy (VWR, Radnor, PA, USA). The mounted samples were sliced with Leica CM 1860 Cryotom (Leica, Nussloch, Germany) and the 6 μm thick slices were prepared for immunohistochemical experiments.

### Immunofluorescence staining and confocal microscopy

B50 neuroblastoma cells were grown on BioCoat Collagen Type I Cellware coverslips (Corning Inc., Corning, NY, USA). Confluent cells were washed with TBS, fixed with ascending alcohol series (70%-80%-96% ethanol for 10–10 min at -20°C) and permeabilized in TBS containing 0.2% Triton X-100. After washing with TBS, cells were incubated in 1% horse serum/TBS (blocking solution) for 1 hour at 4°C and with primary antibodies (diluted in blocking solution at a ratio of 1:100) overnight at 4°C. Coverslips were washed with TBS and incubated with secondary antibodies at 1:200 dilution (Alexa Fluor 488-conjugated anti-rabbit IgG, Alexa Fluor 546-conjugated anti-mouse and anti-goat IgG). Coverslips were mounted in ProLong Gold antifade reagent (Life Technologies, Carlsbad, CA, USA). Cells were examined with a Leica X8 confocal microscope (Leica Microsystems, Wetzlar, Germany). Brain slices were also prepared for immunohistochemistry. Slices were dried, then incubated in blocking buffer consisting of 10% horse serum and 0.2% Triton X-100 in TBS for 1 hour at room temperature and the staining procedure was applied as described above using primary antibodies against SNAP25^pThr138^ and syntaxin.

### Statistical analysis

Normalized data were analysed by either student *t*-test or by ANOVA using GraphPad Prism 6 program and Dunnett’s *t test* as post hoc test All data shown in this article represent mean ±SEM, and *n* means the number of independent experiments. The applied analysis is indicated in the figure legend.

## Results

### Interaction of SNAP-25 and myosin phosphatase regulatory subunit (MYPT1)

**Fig 1:**
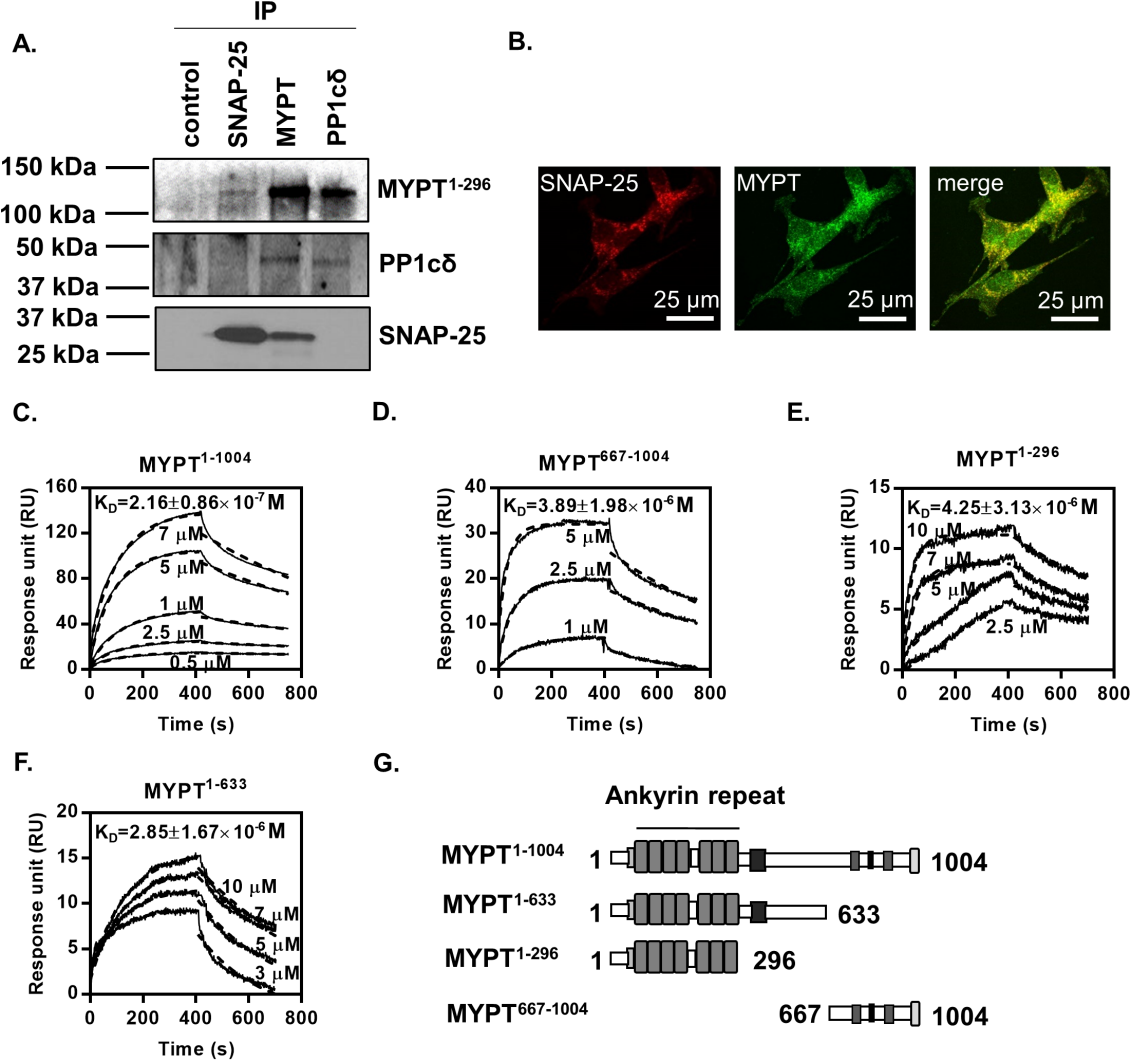
Interaction between SNAP-25 and the MYPT1 subunit of myosin phosphatase. (A) Immunoprecipitation was performed from B50 neuroblastoma cell lysates with antibodies specific for MYPT1, PP1cδ and SNAP-25 coupled to Protein-A Sepharose, and the precipitates were analyzed by Western blots using the indicated antibodies. In control, rabbit non-immune serum was coupled to Protein-A Sepharose. (B) Confocal microscopy of MYPT1 (green) and SNAP-25 (red). Images show the co-localization of SNAP-25 and MYPT1 in a merged image. (C-G) SPR-based binding assays of the interaction between MYPT1 and SNAP-25. Full-length GST-MYPT1^1-1004^ (C) and a C-terminal GST-MYPT1^667-1004^ fragment (D) were immobilized on sensor chips coupled with anti-GST. N-terminal fragments of His-MYPT1^1-296^ (E) and His-MYPT1^1-633^ (F) were immobilized on CM5 sensor chips by direct amine-coupling. Flag-SNAP-25 was injected over the surfaces at the concentrations indicated on the sensorgrams. Dissociation constants (K_D_) derived by the BIAevaluation 3.1 software from the sensorgrams assuming a 1:1 binding model are indicated in the figures. The unit of dissociation constant values is M (n = 3). Schematic structure of the full-length, as well as the N-terminal and C-terminal fragments of MYPT1 used as ligands in the SPR measurements are shown (G).

SNAP-25 was reported as an interacting protein of GST-MYPT1 identified by mass spectrometry [16]. To further study the interaction between SNAP-25 and the subunits of myosin phosphatase, we carried out reciprocal immunoprecipitations (Fig 1A) from B50 neuroblastoma cell lysates using specific anti-SNAP-25, anti-PP1cδ and anti-MYPT1^1-296^ antibodies. The subunits of myosin phosphatase—MYPT1 and PP1cδ—as well as MYPT1 and SNAP-25 were reciprocally co-precipitated, however SNAP-25 and PP1cδ did not show interaction. These data verify the interaction between MYPT1 and SNAP-25, and suggest that the SNAP-25 protein interacts with MP through MYPT1, but not PP1c. Co-localization of SNAP-25 and MYPT in the cytoplasm and neuronal projections of B50 neuroblastoma cells was detected using confocal microscopy (Fig 1B). The Pearson coefficient for MYPT1 and SNAP25 and for GAPDH and MYPT1 (as negative controls of non-interacting proteins) were 0.66 and 0.31, respectively. Similar values were measured in three separate experiments (n = 3), indicating partial but definite co-localization of SNAP-25 with MP in neuronal cells.

To determine the SNAP-25-interacting region of MYPT1, surface plasmon resonance (SPR) experiments were performed with full-length and truncated MYPT1 mutants and Flag-SNAP-25. Full-length wild type MYPT1 (GST-MYPT1^1-1004^) (Fig 1C) and the C-terminal (GST-MYPT1^667-1004^) (Fig 1D) or N-terminal fragments, MYPT1^1-296^ (Fig 1E) and MYPT1^1-633^ (Fig 1F), were immobilized on sensor chip surfaces to assay interaction with SNAP-25 using SPR based binding technique. The schemes of these MYPT1 mutants are shown in Fig 1G. Based on the sensorgrams obtained by injection of SNAP-25 over these MYPT1 surfaces a relatively strong interaction with the full-length MYPT1 (K_D_ = 2.16±0.86x10^-7^) was detected (Fig 1C). It was also apparent that SNAP-25 binds to both the C-terminal (K_D_ = 4.25±3.13x10^-7^) and N-terminal (MYPT1^1-296^: K_D_ = 3.89±1.98x10^-6^; MYPT1^1-633^: K_D_ = 2.85±1.67x10^-6^) regions of MYPT1 with preference for the C-terminal region, based on the derived dissociation constants.

### Phosphorylation/dephosphorylation of SNAP-25 by ROK/MP

**Fig 2:**
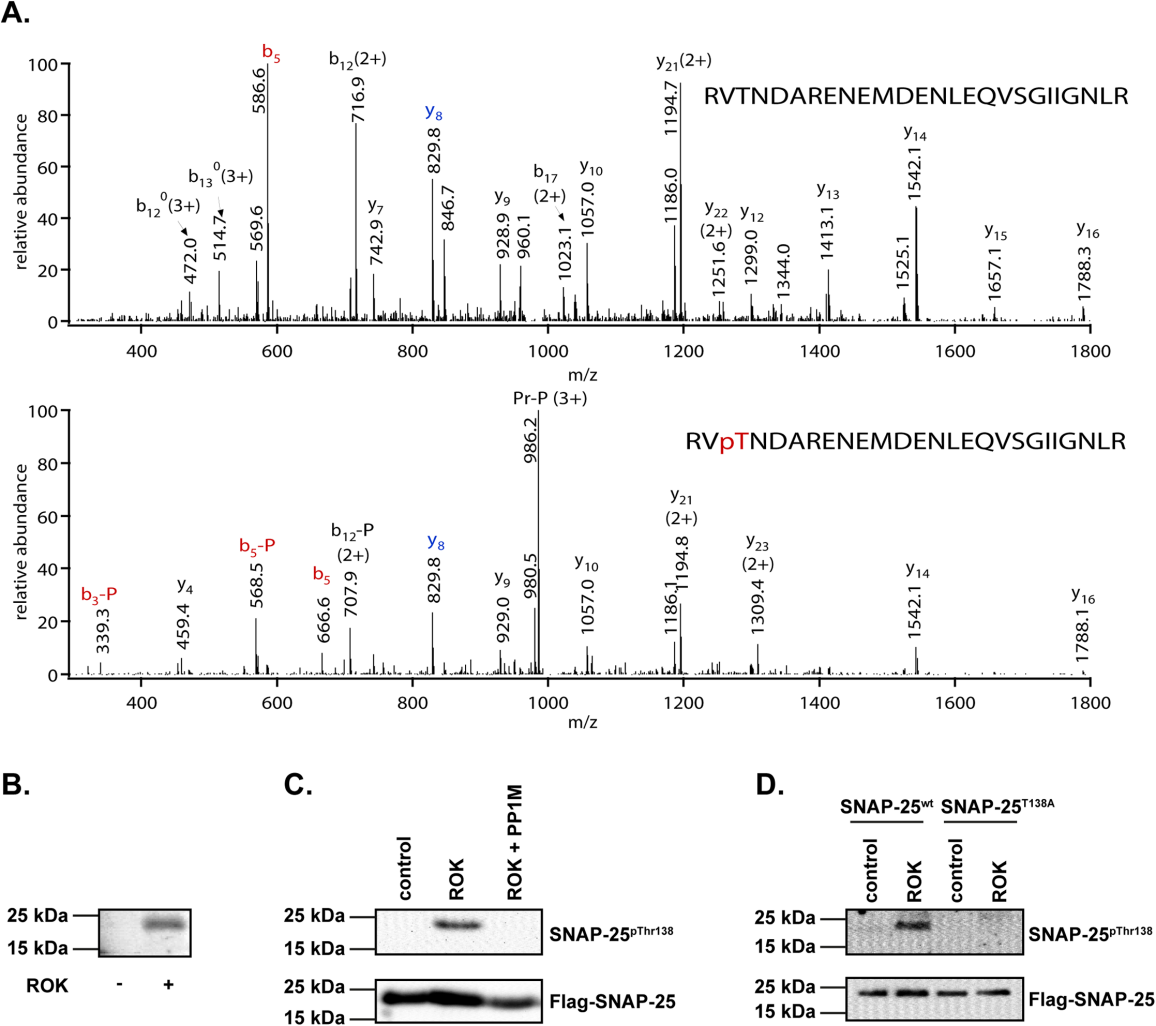
Phosphorylation and dephosphorylation of purified Flag-SNAP-25. (A) Mass spectrometry analysis of ROK-phosphorylated SNAP-25. Collision induced dissociation (CID) spectra representing SNAP-25 [136–161] peptides. Upper panel: m/z: 991.4868 (3+), ^136^RVTNDARENEMDENLEQVSGIIGNLR^161^. Lower panel: m/z: 1018.1437 (3+), ^136^RVT(Phospho)NDARENEMDENLEQVSGIIGNLR^161^. Site of phosphorylation is Thr-138 as proven by unmodified y_8_ and phosphorylated b_5_ fragment ions. Peptide fragments are labeled according to the nomenclature [34]. -P stands for the 98-Da neutral loss of phosphoric acid characteristic to Ser/Thr phosphorylation, pr stands for the precursor ion and ^0^ denotes water loss of the respective fragment ions. (B) Phosphorylation of SNAP-25 by RhoA-activated kinase (ROK) in the presence of γ-^32^P-ATP and Mg^2+^ as detailed in Materials and Methods. No ROK was introduced to control samples. Phosphorylation was detected by autoradiography. (C) Examination of SNAP-25 phosphorylation/dephosphorylation on Thr138 by *in vitro* kinase and phosphatase assays. Flag-SNAP-25 was incubated in the absence (control) or presence of ROK and then with or without MP. Phosphorylation level of Thr138 was investigated by Western blot using anti-SNAP25^pThr138^ phosphorylation site-specific antibody. The loading of Flag-SNAP-25 was verified by anti-Flag antibody. (D) *In vitro* protein kinase assay of wild type (SNAP-25^wt^) and Thr138Ala mutant (SNAP-25^T138A^) of SNAP-25 by ROK. The level of SNAP-25^pThr138^ was assessed by anti-SNAP-25^pThr138^ antibody by Western blot.

Previous results have shown that RhoA-activated kinase (ROK) and MP have some common neuronal substrates, e. g. synapsin-I, syntaxin-1, and the 20 kDa light chain of myosin-II [16]. This fact motivated us to study not just the role of MP, but also the role of ROK, in the regulation of SNAP-25 by phosphorylation. To identify the potential ROK-specific phosphorylation sites, Flag-SNAP25 was phosphorylated by ROK and was subjected to LC-MS/MS analysis after tryptic digestion (Fig 2A). The following SNAP-25 Thr138 related phosphopeptides were identified in the samples: ^136^RVT(phospho)NDAR^142^, ^136^RVT(phospho)NDARENEMDENLEQVSGIIGNLR^161^, and ^136^RVT(phospho)NDARENEM(oxidation)DENLEQVSGIIGNLR^16^^1^. All of these peptides were identified in the ROK treated sample, while only the latter one was identified in the control sample (S2 Table). The phosphorylation of Thr138 residue of SNAP-25 was highly elevated in the sample in the presence of ROK. Comparison of the ion intensities of m/z 1023.4749, representing the most abundant Thr138 related phosphopeptide indicated an approximately 100-fold increase compared to the control sample. ROK phosphorylation was also verified by ^32^P-phosphate incorporation into SNAP-25 detected by autoradiography (Fig 2B.). Flag-SNAP-25 protein was also phosphorylated by ROK and the ROK-phosphorylated Flag-SNAP25 was subjected to dephosphorylation by myosin phosphatase using i*n vitro* assays (Fig 2C). The phosphorylation state of SNAP-25 on Thr138 was analyzed by Western blot using anti-SNAP25^pThr138^ antibody. Increased phosphorylation of Thr138 was observed in response to ROK treatment compared to control samples. The addition of MP followed by ROK eliminated the phosphorylation of SNAP-25 at Thr138 residue. To verify the specificity of the phosphorylation by ROK, Thr138Ala mutant SNAP-25 (SNAP-25^T138A^) was generated by site-directed mutagenesis and the wild type (SNAP-25^wt^) and mutant SNAP-25 were incubated with ROK. Phosphorylation was analyzed by Western blots using anti-SNAP-25^pThr138^ antibody (Fig 2D). It was apparent that only the Flag-SNAP-25^wt^ but not the Flag-SNAP-25^T138A^ was phosphorylated by ROK. These experimental data suggest that ROK phosphorylates and MP dephosphorylates SNAP-25 on the Thr138 residue *in vitro*.

### Inhibition of ROK and MP influences the phosphorylation level of SNAP-25 at Thr138 in B50 neuroblastoma cells

**Fig 3:**
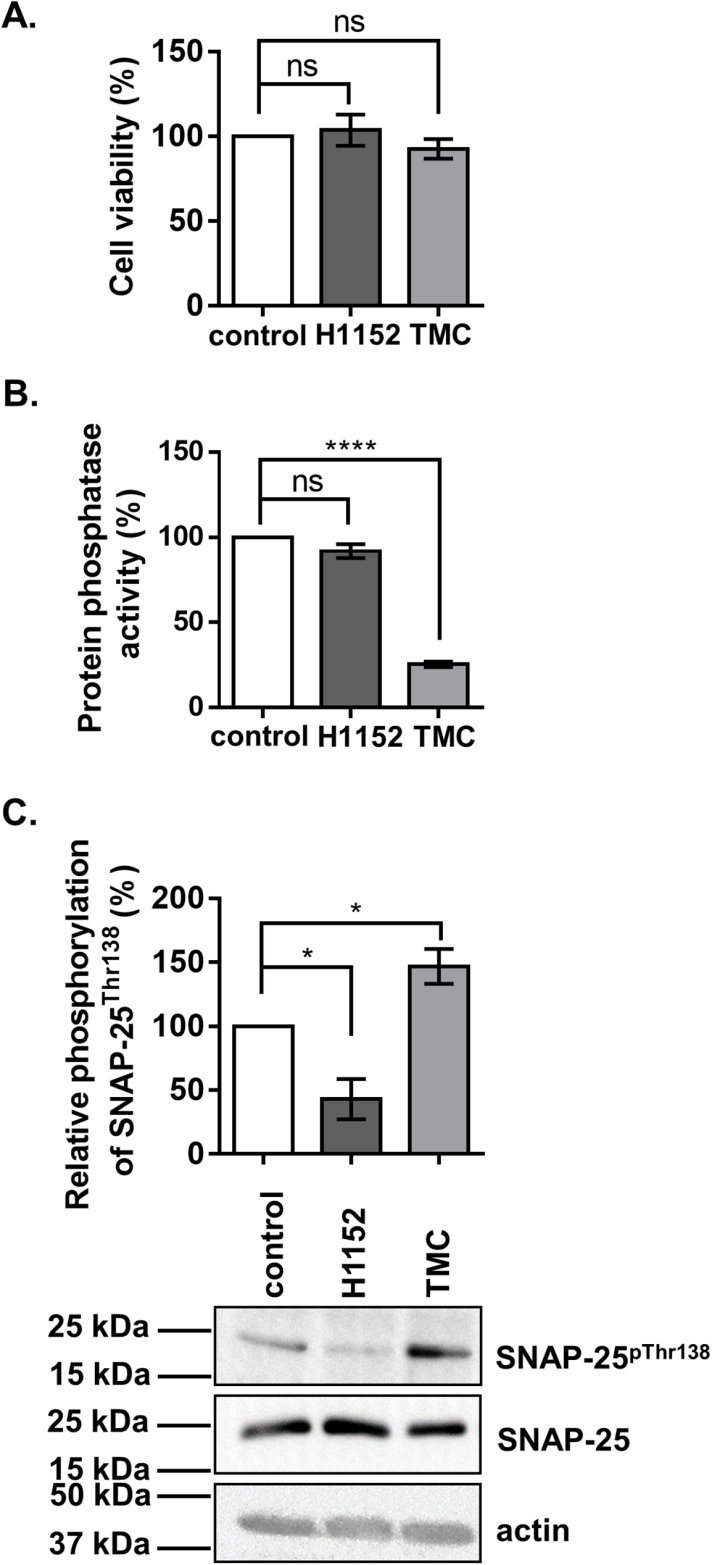
Treatment of B50 neuroblastoma cells with PP1 and ROK inhibitors. B50 cells were incubated with tautomycetin (TMC), a PP1 specific inhibitor, or H1152, a ROK inhibitor, for 1 hour and 30 minutes respectively. Untreated cells served as controls. (A) Viability of B50 cells were examined using MTT assay. Values are mean±SEM (n = 6), p = 0.4645. (B) Effect of inhibitors on protein phosphatase activity. Values are mean±SEM (n = 3), p<0.0001. (C) The effect of the inhibitory treatment on the phosphorylation level of Thr138 in SNAP-25 was assessed by Western blots using anti-SNAP25^pThr138^. Values are mean±SEM (n = 3), p = 0.0027. Amounts of SNAP-25 and actin were also determined as loading controls. Relative changes in the phosphorylation levels were determined by densitometry of the bands. Means ±SEM (n = 5), ANOVA and Dunnett’s post hoc analysis, *p<0.05; **p<0.01; ***p<0.001; ****p<0.0001.

To prove the *in vivo* regulatory effect of ROK and MP on SNAP-25, B50 neuroblastoma cells were treated with 10 μM H1152, a ROK-specific inhibitor, or 5 μM tautomycetin (TMC), a PP1-specific inhibitor. The inhibitors had no significant effect on the viability of B50 cells measured by MTT assay (Fig 3A). The protein phosphatase activity was determined using ^32^P-labelled 20 kDa myosin light chain (^32^P-MLC20) as substrate. Upon inhibition of PP1 with TMC the protein phosphatase activity dramatically decreased compared to the control untreated samples, suggesting that PP1 is responsible for approximately 70% of the total protein phosphatase activity in B50 cells. H1152 treatment had no significant effect on the phosphatase activity (Fig 3B). The changes in SNAP-25 Thr138 phosphorylation levels in response to inhibitory treatments were examined by Western blot using anti-SNAP-25^pThr138^. Inhibition of ROK by H1152 decreased the phosphorylation at SNAP-25^pThr138^ by 57% compared to control, while PP1 inhibition resulted in an increase in SNAP-25^pThr138^ level to 147% (Fig 3C).

### Silencing MYPT1 increases the phosphorylation level at Thr138 of SNAP-25

**Fig 4:**
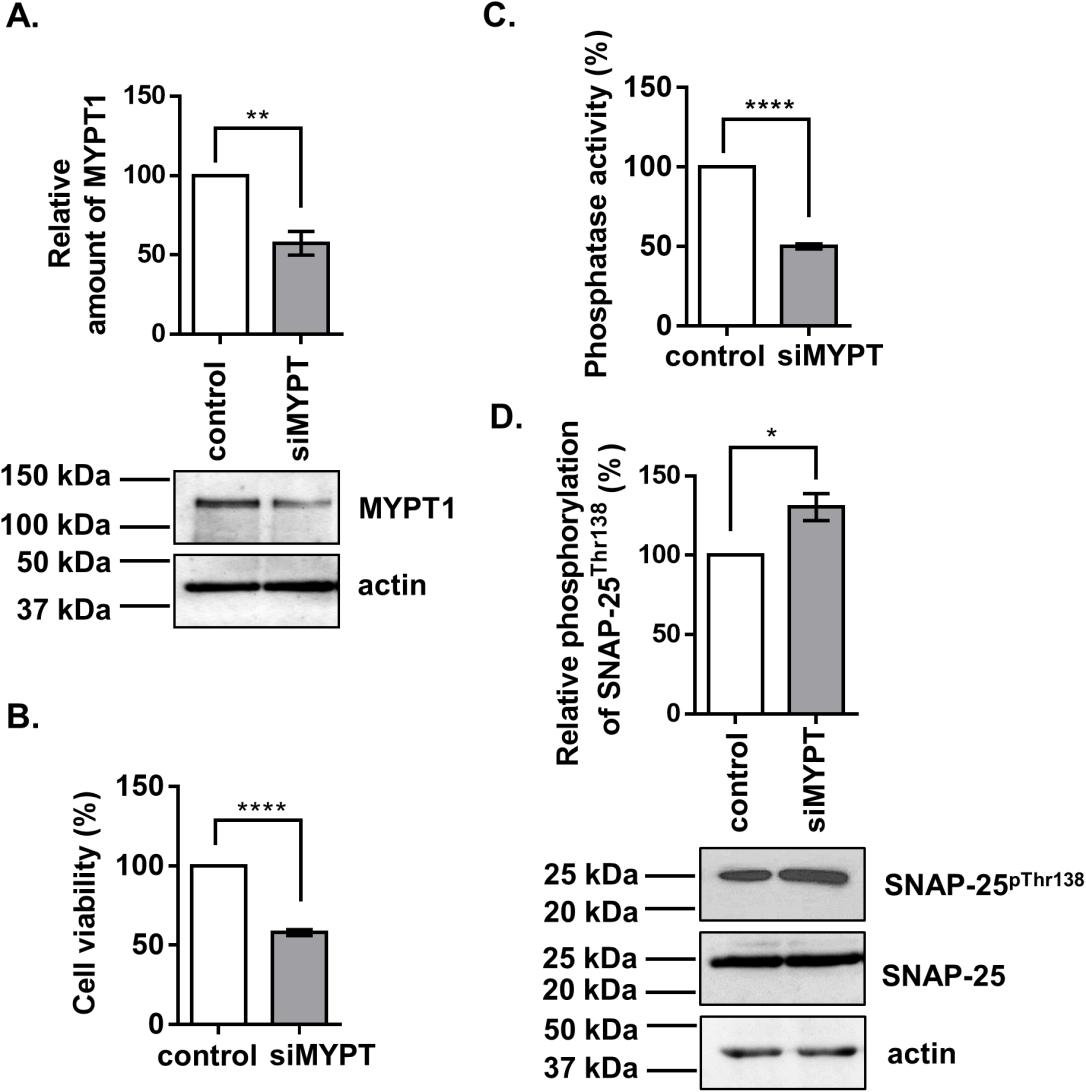
Silencing of MYPT1 by siRNA in B50 neuroblastoma cell line. (A) Western blot images of cell lysates after treatments with 100 nM of siRNA targeting the endogenous MYPT1 subunit. The relative amount of MYPT1 was detected and determined with anti-MYPT1^1-296^ antibody. (B) MTT cell viability assays were carried out 48 hours after transfecting cells with MYPT1 specific siRNA. (C) Assay of phosphatase activity in control and siRNA transfected cell lysates with ^32^P-MLC20 substrate. (D) The phosphorylation of SNAP-25 on Thr138 was detected by anti-SNAP25^pThr138^, and the levels of SNAP-25 as well as actin from control and transfected cells was also determined. The changes in the level of the phosphorylated SNAP-25 was determined comparing the relative density of the bands normalized for the loading controls. Means ±SEM (n = 5), student t test, *p<0.05; **p<0.01;; ****p<0.0001.

The fact that PP1 inhibitors influence the phosphorylation of SNAP-25 at Thr138 was proven; however, the contribution of MP inhibition to this effect remained unclear. To assess the regulatory role of MP on the dephosphorylation of SNAP-25, gene silencing of the MYPT1 regulatory subunit in B50 cells was carried out. The extent of MYPT1 silencing was verified by Western blot analysis and 43% decrease in the protein expression level of MYPT1 was detected compared to non-target controls (Fig 4A). Knocking down MYPT1 showed a 42% decrease in the viability of B50 cells compared to control as examined by MTT assay (Fig 4B). Silencing of MYPT1 showed a 50% decrease in overall phosphatase activity (Fig 4C) suggesting that MP is a predominant PP1 enzyme in B50 neuroblastoma cells. In response to knocking down MYPT1, the SNAP-25 phosphorylation on Thr138 significantly increased by 30% compared to control (Fig 4D) as assessed by semi-quantitative Western blots. This suggests that MP dephosphorylates SNAP-25 on Thr138 in B50 neuroblastoma cells and this dephosphorylation can be diminished by silencing of MYPT1, the targeting subunit of the dephosphorylation process.

### MP increases the exocytosis of mouse cortical synaptosomes through the dephosphorylation of SNAP-25

**Fig 5:**
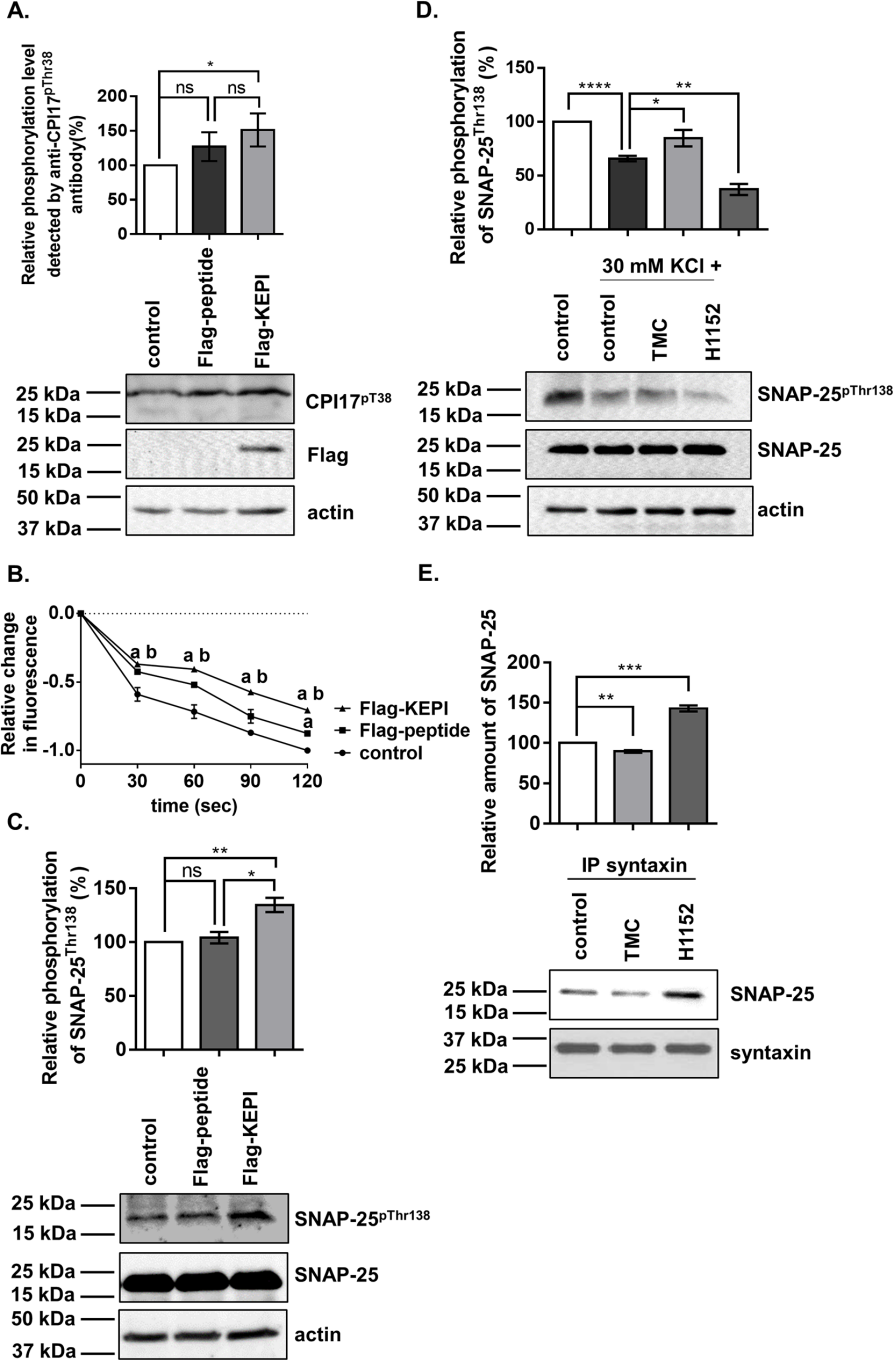
Effect of PP1 and ROK inhibitors on the phosphorylation level of SNAP-25 at Thr-138 and on exocytosis in mouse cortical synaptosomes. (A) Flag-KEPI, a myosin phosphatase specific inhibitor protein, was introduced into the synaptosomes by the freeze-thaw method. To distinguish the effect of Flag-tag from Flag-KEPI, Flag-peptide was also introduced. Control synaptosomes underwent the freeze-thaw process, but no protein or peptide was added. Transduction of Flag-KEPI into synaptosomes was verified by western blot analysis using anti-Flag antibody. Relative phosphorylation levels were detected by anti-CPI17^pThr38^ antibody. C-kinase activated phosphatase inhibitor protein of 17 kDa (CPI17) and KEPI are homologous MP inhibitor proteins; both need phosphorylation to become active. As the surroundings of the phosphorylation sites of CPI17 and KEPI are similar, anti-CPI17^pThr38^ antibody can recognize the phosphorylated, active KEPI protein. ANOVA: p = 0.0389. (B) Changes in the extent of exocytosis in response to Flag-peptide or Flag-KEPI transduction was measured by incorporating FM 2–10 fluorescent dye into the synaptosomes, and determining the release of dye. Each point represents the mean±SEM of three parallel experiments (n = 3). Symbols: *a*: significant (p<0.05) compared to control; *b*: significant compared to Flag-peptide; t-test was used. By ANOVA, we detected significant differences among treated groups (p = 0.006). (C) Effects of Flag-peptide and Flag-KEPI transduction on the phosphorylation of SNAP-25 at Thr138. ANOVA: p = 0.0050. (D) Effect of KCl depolarisation, in the absence and presence of TMC or H1152 treatments, on the phosphorylation level of Thr138 in SNAP-25 analysed by western blots using anti-SNAP25^pThr138^. The duration of treatment was 1 hour. Actin was assessed as loading control. ANOVA: p < 0.0001. (E) Effect of TMC and H1152 treatment of synaptosomes on the interaction of SNAP-25 and syntaxin. Synaptosomes were treated with the inhibitors as mentioned above then lysed. Immunoprecipitations from synaptosome lysates were performed using anti-syntaxin antibody. The amount of Protein-A Sepharose was the same in each tube. The amounts of precipitated syntaxin and SNAP-25 were detected by Western blots. ANOVA: p<0.0001. Statistical analyses were performed using the data of n = 3–5 parallel experiments and representative blots are shown here. Means ±SEM (n = 5), ANOVA and Dunnett’s post hoc analysis, *p<0.05; **p<0.01; ***p<0.001; ****p<0.0001.

Previous experiments have shown that treatment with the PP1 inhibitor, TMC, suppressed, whereas the ROK inhibitor, Y27632, increased, the level of Ca^2+^-dependent exocytosis from rat cortical synaptosomes [16]. To determine the physiological role of SNAP-25 regulation by MP and ROK in neurotransmitter release, functional synaptosomes were prepared from mouse cortex and used as models in a high-throughput exocytosis assay. The MP-dependent physiological responses were assayed by protein transduction of a Flag-tagged kinase enhanced phosphatase inhibitor (Flag-KEPI) which specifically inhibits MP among the PP1 holoenzymes [33]. The transduction of Flag-KEPI into synaptosomes was verified by Western blot analysis using anti-Flag antibody (Fig 5A). Since the phosphorylation of KEPI at Thr73 is required for its inhibitory activity [35] the phosphorylation status was determined by using an antibody specific for the phosphorylated Thr38 of C-kinase activated phosphatase inhibitory protein of 17 kDa (CPI-17) which also recognizes the phosphorylation of KEPI^Thr73^ [29]. The transduction of Flag-KEPI into intact synaptosomes increased the phosphorylation level of KEPI compared to both the intact control and the Flag-peptide transduced samples (Fig 5A) suggesting the inhibitory potential of KEPI on MP. The basal phosphorylation in control and Flag-introduced synaptosomes is suspected to be derived from the endogenous KEPI (Fig 5A). KCl-induced exocytosis was investigated in Flag-KEPI transduced synaptosomes. The synaptosomes were loaded with FM 2–10 fluorescent dye and the reduction of fluorescence intensity was recorded every 30 seconds for 3 minutes. While transduction of Flag-peptide had no significant effect on exocytosis at any time point, Flag-KEPI transduction decreased the exocytosis by 28–39% compared to untreated controls (Fig 5B) suggesting an activating role of MP in neurotransmitter release. The most significant changes were observed after the first 30 sec of the measurement. To clarify the molecular mechanism behind the physiological processes regulated by MP, the phosphorylation status of SNAP-25 on Thr138 in response to Flag or Flag-KEPI transduction was investigated by Western blot analysis. The basal level of SNAP-25^pThr138^ was not altered by the transduction of the Flag peptide, but it was significantly increased (by 34%) upon the inhibition of MP by Flag-KEPI transduction (Fig 5C). The effect of depolarization on SNAP-25^pThr138^ level was assessed in synaptosomes by Western blots (Fig 5D). The relative phosphorylation at Thr138 of SNAP-25 upon addition of KCl was lower than in the untreated controls suggesting that depolarization itself has an influence on the phosphorylation status of SNAP-25. After depolarization with KCl, the basal level of phosphorylation on Thr138 was increased in the presence of TMC, and significantly decreased by addition of H1152. Based on our previous results, we attempted to verify the effect of SNAP-25 phosphorylation at Thr138 on the SNARE complex formation by immunoprecipitation assay using an antibody specific for syntaxin, a member of the SNARE complex, in TMC or H1152-treated synaptosome lysates (Fig 5E). The relative amount of SNAP-25 in the anti-syntaxin pulled down fractions decreased upon PP1 inhibition, whereas it increased following ROK inhibition suggesting that decreasing phosphorylation level of SNAP-25 in Thr138 favors interaction between SNAP-25 and syntaxin.

### ROK and MP mediate the phosphorylation level of SNAP-25^Thr138^ in brain slices

**Fig 6:**
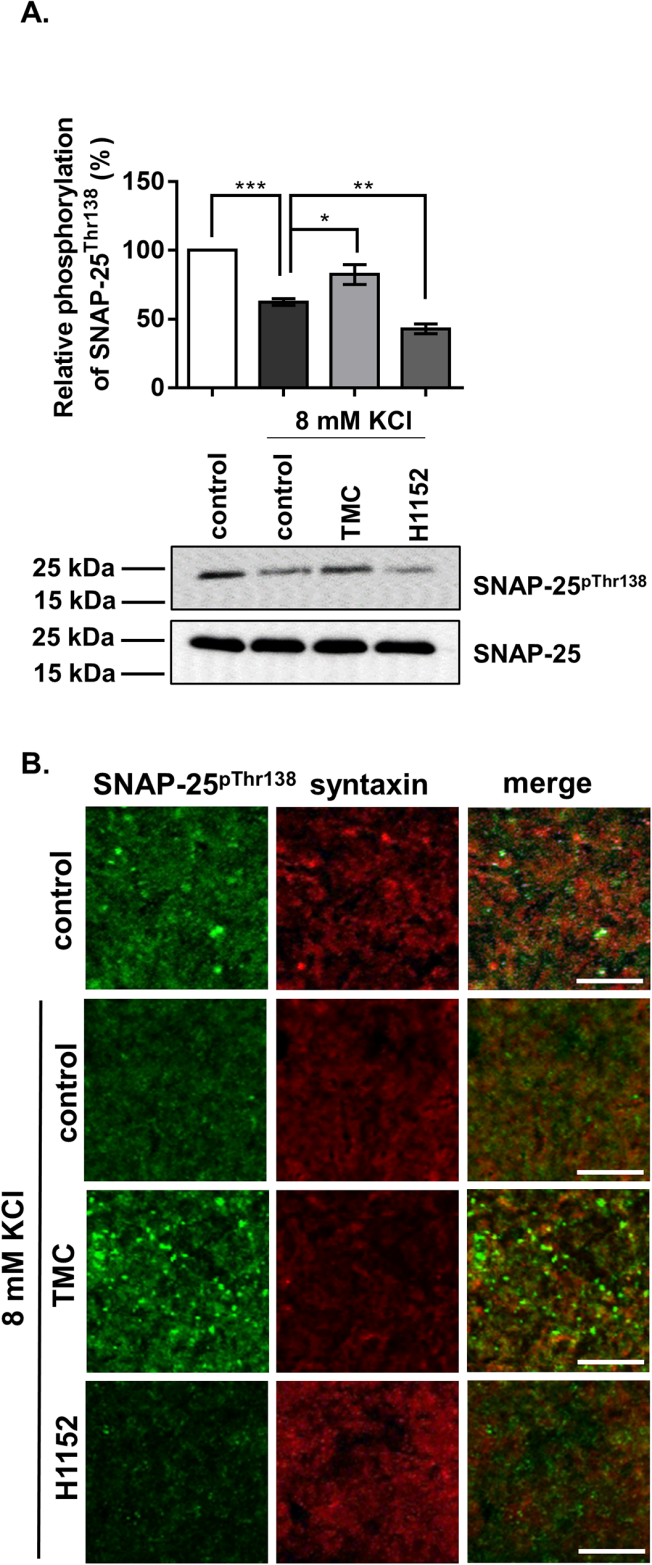
Effect of PP1 and ROK inhibitors on the phosphorylation of SNAP-25 at Thr138 in brain slices. (A) Mouse cortical brain slices were prepared and treated with TMC or H1152 for 1 hour. To trigger depolarization, 8 mM KCl was added. Phosphorylation of SNAP-25 was investigated by Western blot using an anti-SNAP-25^pThr138^ antibody. Amounts of SNAP-25 protein were also determined by anti-SNAP-25 antibody. Density of bands wase determined and normalized relative phosphorylation is illustrated. Means ±SEM (n = 5), ANOVA and Dunnett’s post hoc analysis, *p<0.05; **p<0.01; ***p<0.001. (B) Changes in SNAP-Thr138 phosphorylation in response to treatments as detailed in (A) were also imaged by confocal microscopy. The four sets of brain slices (control, KCl-treated, KCl + TMC treated, KCl + H1152 treated) were analysed with anti-SNAP-25^pThr138^ antibody to assess differences in phosphorylation level, and also with anti-syntaxin antibody to identify syntaxin, a typical pre-synaptic protein. Co-localizations of syntaxin and phospho-SNAP-25 are also shown on merged images. Scale bar: 10 μm.

To confirm the previously described results obtained with synaptosomes on an *ex vivo* physiological model, we performed experiments using brain slices prepared from murine cortex maintained in low-Na^+^ artificial cerebrospinal fluid and depolarized by KCl in a solution containing TMC (5 μM) or H1152 (10 μM). First, the expression and phosphorylation level of SNAP-25 at Thr138 were examined. Supporting our previous observations with synaptosomes, the results in brain slices show that inhibition of PP1 by TMC increased, whereas inhibition of ROK by H1152 decreased, the level of SNAP-25^pThr138^. Moreover, KCl-induced depolarization also caused a significant decrease in the level of SNAP-25^pThr138^ (Fig 6A). We visualized the changes of SNAP-25^pThr138^ (green) by confocal microscopy, and syntaxin (red) was also labeled as a presynaptic marker. As seen in Fig 6B, stimulation by 8 mM KCl decreased the overall SNAP-25 phosphorylation at Thr138 without influencing the expression of syntaxin. Inhibition of PP1 with TMC increased, whereas the application of H1152 almost completely eliminated phosphorylation, at Thr138 of SNAP-25 upon KCl-depolarization (Fig 6B).

## Discussion

Several lines of evidences suggest that the neuronal exocytosis is regulated by the phosphorylation of the membrane fusion machinery of the SNARE complex [36, 37]. However, the precise roles of individual phosphorylation events of specific proteins are still contradictory. The SNARE-complex protein, SNAP-25 has been previously reported to be modulated by phosphorylation on multiple sites upon physiological responses [38, 39]. In this study, we identified SNAP-25 as a novel substrate of the ROK and MP enzymes and its phosphorylation at Thr138 correlates with changes in neuronal exocytosis of murine cortical synaptosomes. This work extends our previous reports showing that MP and ROK are present in both cortical synaptosomes and in giant synapses of rat auditory brain stem, and that they influence synaptosomal exocytosis and neurotransmitter release in an opposing manner: PP1 inhibition suppresses, whereas ROK inhibition stimulates exocytosis [16].

In search for the common targets of ROK/MP, mass spectrometry analysis identified several synaptosomal MYPT1-interacting proteins such as synapsin-I, calcineurin-A subunit, Ca^2+^-calmodulin dependent kinase II, as well as syntaxin-1 and SNAP-25 of the SNARE complex [16]. Here we confirm by co-immunoprecipitation that SNAP-25 interacts with the MYPT1 regulatory subunit of MP (Fig 1A). An SPR-based *in vitro* binding assay identified a stable interaction between MYPT1 and SNAP-25 which verified that SNAP-25 binds to both the N-terminal and C-terminal regions of MYPT1 (Fig 1C–1F).

Our findings show that ROK and MP counteract each other at the Thr138 phosphorylation site of SNAP-25 *in vivo* and *in vitro* (Figs 2–4). Previous experiments showed that SNAP-25 is phosphorylated by both PKA and PKC at residues Ser28, Thr29, Thr138, and Ser187, but PKC showed higher preference toward the latter two over Ser28 and Thr29 [15, 38, 40, 41]. A recently published study showed that Thr138 of SNAP-25 is phosphorylated selectively by PKA upon ATP treatment of PC12 cells, whereas Ser187 of SNAP-25 is phosphorylated preferentially by PKC, suggesting different roles of PKA and PKC in SNAP-25 phosphorylation [42]. Here we report that ROK is a novel regulator of SNAP-25. ROK phosphorylates SNAP-25 at the Thr138 residue, but not at Ser28, Thr29, or Ser187, as revealed by mass spectrometry analysis. These data suggest a RhoA-dependent regulation of the SNARE complex through the Thr138 site of SNAP-25.

The inhibition of PP1 by TMC increased the phosphorylation of SNAP-25 at Thr138 in B50 neuroblastoma cells (Fig 3), in KCl-depolarized cortical synaptosomes (Fig 5), and in cortical thin brain slices (Fig 6). These findings also support the already well-described role of PP1 enzymes in the regulation of SNARE complex formation [43, 44]. SNAP-25 phosphorylated by PKA and PKC was dephosphorylated more efficiently by PP1 and to a lesser extent by PP2A but not by PP2B *in vitro* [42]. PP1 enzymes act equally efficiently at the Ser28, Thr 29, Thr138, and Ser187 residues of SNAP-25, and PP1 is the major phosphatase responsible for dephosphorylating SNAP-25 [45]. Furthermore, SNAP-25 was dephosphorylated at Thr138 by MP (the holoenzyme of PP1c and MYPT1) (Fig 2) and the selective inhibition of MP by KEPI enhanced the Thr138 phosphorylation (Fig 5C). The above data are in accordance with our previous findings that MP is the predominant PP1 holoenzyme in synaptosomes and presynaptic cells suggesting that MP is a major determinant of PP1-dependent dephosphorylation processes [20].

The final step of exocytosis, membrane fusion, is regulated by the phosphorylation of SNARE and accessory proteins. A large body of evidence show that SNAP-25 phosphorylation by PKA or PKC contributes differentially and selectively to the control of exocytosis in PC12 cells by regulating the SNARE complex formation [13, 45, 46]. Phosphorylation of SNAP-25 at The Ser187 was suggested to upregulate the binding of synaptobrevin to a pre-existing t-SNARE to increase the stability of the SNARE complex. Indeed, it was reported that PKC activation initiated an enhanced K^+^-induced noradrenaline release in PC12 cells. [42]. Moreover, the phosphorylation of SNAP-25 at Ser187 by PKC was necessary for the enhancement of Ca^2+^-triggered exocytosis by phorbol ester in an insulinoma cell line [47], and the inhibition of SNAP-25 phosphorylation at Ser187 down-regulated morphine-induced SNARE complex formation in neurons [48].

In contrast to Ser187 phosphorylation, phosphorylation of SNAP25 at Thr138 may contribute to the suppression of the SNARE complex formation [38]. Thr138 is part of the peptide region in SNAP-25 that influences SNARE complex assembly and the binding of SNAP-25 to syntaxin-1. Thus, the formation of SNARE complex was suppressed both by PKA-treatment of the purified SNAP-25 and forskolin treatment of PC12 cells [42]. Moreover, forskolin stimulation of PKA resulted in a dramatic phosphorylation of SNAP^pThr138^ without appreciable changes in neurotransmitter release [15]. On the contrary, the activation of PKA indirectly enhanced the Ca^2+^-dependent exocytosis by maintaining the large number of vesicles in the ready-to-release pool in chromaffin cells. However, the overexpression of the T138D phosphomimic form of SNAP-25 did not modify the secretion significantly [14, 49]. It was also clarified that the phosphorylation of SNAP-25 at Thr138 inhibits noradrenaline secretion of differentiated PC12 pheochromocytoma cells [42].

Several bodies of evidence indicate the importance of ROK-dependent phosphorylation in regulated exocytosis via interference with SNARE functions. In chromaffin cells and neurons, the RhoA/ROK pathway governs the secretory response by phosphorylating syntaxin 1 and facilitating the binding of syntaxin 1 to tomosyn, thereby impairing SNARE complex formation and contributing to reduced exocytosis [50, 51]. Our results clearly confirm that the binding of SNAP-25 to syntaxin-1 and the formation of the SNARE complex were inhibited by TMC- and elevated by H1152-treatment of synaptosomes. The increase in syntaxin-SNAP25 complex formation was accompanied by the decrease of the phosphorylation of SNAP-25 at Thr138 (Fig 5E). We also observed that the modulation of the Thr138 site, as a result of the inhibition of ROK by H1152 [16] and the inhibition of MP by KEPI protein, positively and negatively regulates synaptosomal exocytosis (Fig 5), respectively. These data indicate that ROK and MP may modulate exocytosis by directly modifying the SNARE and docking complex functions. Since the most dramatic change in KCl-induced exocytosis in control and Flag-KEPI transduced synaptosomes was observed in the first 30 seconds after depolarization, we suggest that the inhibition of myosin phosphatase predominantly impacts the formation of the SNARE complex and the process of priming rather than the fusion itself. In this study, we also clarified that selective inhibition of MP by KEPI protein suppresses KCl-induced synaptosomal exocytosis (Fig 5B). These data are reminiscent of our previous data showing that PP1 and ROK inhibition affected synaptic transmission in giant synapse in the rat auditory system; PP1 inhibition decreased, whereas ROK-inhibition increased, the release probability. Moreover, the inhibition of PP1 changed the paired-pulse ratio and it caused a significant reduction in the frequency of the relative amplitudes of the third and subsequent evoked miniature excitatory post-synaptic currents [16]. These results may be explained by the fact that several proteins related to exocytosis in intact synaptosomes are substrates of ROK and MP, including MYPT1^Thr696^, myosin-II light chain^Ser19^, synapsin-I^Ser9^ and syntaxin-1^Ser14^, and the overall effect of MP and ROK on the phosphorylation status of these presynaptic proteins regulates neurotransmission. The other possible explanation is that under our experimental conditions with PP1 and ROK inhibitors, the applied [Ca^2+^] was already high (1.2 mM, 1.8 mM and 2 mM in synaptosomal buffer, DMEM and in aCSF, respectively) which may prevent the action of PKA on SNAP-25 and on other presynaptic targets as described on chromaffin cells [14]. The RhoA signaling pathway and MP may also play a role in the regulation of SNAP-25 at Thr138 and other potential synaptosomal targets in a Ca^2+^ independent way.

In conclusion, we have shown that ROK phosphorylates and MP dephosphorylates SNAP-25 at Thr138 in synaptosomes, cortical thin slices and B50 neuroblastoma cells to regulate the assembly of the SNARE complex and neuronal exocytosis. Our findings provide an important insight into the fine-tuned balance of the action of protein kinases and phosphatases regulating neurotransmitter release in response to changes in physiological conditions.

## Supporting information

S1 Table Site-directed mutagenesis of SNAP25 primer pair. (DOCX)

S2 Table siRNA sequences for MYPT1 silencing. (DOCX)

S3 Table Thr-138 related phosphopeptides identified from LC-MS/MS data of the tryptic digest of control and ROK-treated SNAP-25. (DOCX)

S4 Table NC3Rs ARRIVE guidelines checklist horvath. (PDF)

## Abbreviations

PP1: protein phosphatase-1
PP1c: PP1 catalytic subunit
MP: myosin phosphatase
MYPT1: myosin phosphatase target subunit-1
ROK: RhoA-activated kinase
TMC: tautomycetin
SNARE: soluble N-ethylmaleimide sensitive factor attachment receptor
SNAP-25: 25 kDa synaptosomal associated protein
PKA: protein kinase A
PKC: protein kinase C
CPI-17: C-kinase activated phosphatase inhibitor protein of 17 kDa
KEPI: kinase-enhanced PP1 inhibitor
H1152: (S)-(+)-2-methyl-1-[(4-methyl-5-isoquinolinyl)sulfonyl]homopiperazine∙2HCl
Y27632: *trans*-4-[(1R)-1-aminoethyl]-*N*-4-pyridinylcyclohexane-carboxiamide dihydrochloride
SDS-PAGE: sodium dodecyl sulfate-polyacrylamide gel electrophoresis
PBS: phosphate buffered saline
TBS: Tris-buffered saline solution
TBST: Tris-buffered saline solution containing 0.1% Tween-20
aCSF: artificial cerebrospinal fluid
FBS: fetal bovine serum
BSA: bovine serum albumin
PAS: protein-A Sepharose
MC-LR: microcystin-LR
IP: immunoprecipitation
PEI: polyethylenimine
DMEM: Dulbecco’s modified Eagle’s medium
RIPA: radioimmunoprecipitation assay
TCA: trichloroacetic acid
SPR: surface plasmon resonance
