# Correction: Myosin phosphatase and RhoA-activated kinase modulate neurotransmitter release by regulating SNAP-25 of SNARE complex

**DOI:** 10.1371/journal.pone.0179296

**Published:** 2017-06-05

**Authors:** Dániel Horváth, István Tamás, Adrienn Sipos, Zsuzsanna Darula, Bálint Bécsi, Dénes Nagy, Judit Iván, Ferenc Erdődi, Beáta Lontay

The statement in the Funding section is incorrect. The correct funding information is as follows: This work was supported by grants from the Hungarian Scientific Research Fund OTKA PD104878 and K109249, and by TÁMOP 4.2.2.A‐11/1/KONV‐2012‐0025, TÁMOP‐4.2.2/B‐10/1‐2010‐0024, and TÁMOP‐4.2.4.A/2‐11‐1‐2012‐0001 projects, and by the University of Debrecen (RH/751/2015).
