# Supplementary material for: Myosin phosphatase and RhoA-activated kinase modulate neurotransmitter release by regulating SNAP-25 of SNARE complex
Source: PLoS One. 2017 May 9;12(5):e0177046. doi: 10.1371/journal.pone.0177046 (PMC5423623; doi:10.1371/journal.pone.0177046)
Supplement: S1 Table — (DOCX) [file pone.0177046.s001.docx]

**Table S1. Site-directed mutagenesis of SNAP25 primer pair**

| SNAP25 mutant | Forward primer (5’ to 3’) | Reverse primer (5’ to 3’) |
| --- | --- | --- |
| SNAP25^T138A^ | CCT CAT CCG CAG GGT AGC AAA TGA TGC CCG AGA | TCT CGG GCA TCA TTT GCT ACC CTG CGG ATG AAG |
