# Supplementary material for: Myosin phosphatase and RhoA-activated kinase modulate neurotransmitter release by regulating SNAP-25 of SNARE complex
Source: PLoS One. 2017 May 9;12(5):e0177046. doi: 10.1371/journal.pone.0177046 (PMC5423623; doi:10.1371/journal.pone.0177046)
Supplement: S2 Table — (DOCX) [file pone.0177046.s002.docx]

**Table S2. siRNA sequences for MYPT1 silencing**

siRNA #1: 5’ – CAACUAAACAGGCCAAAUA – 3’

siRNA #2: 5’ – GCUAAAUAGUGGUCAUAUA – 3’

siRNA #3: 5’ – ACAAAGAGACGUUGAUUAU – 3’

siRNA #4: 5’ – CGGAUUCCAUUUCUAGAUA – 3’
