# Supplementary material for: Myosin phosphatase and RhoA-activated kinase modulate neurotransmitter release by regulating SNAP-25 of SNARE complex
Source: PLoS One. 2017 May 9;12(5):e0177046. doi: 10.1371/journal.pone.0177046 (PMC5423623; doi:10.1371/journal.pone.0177046)
Supplement: S3 Table — (DOCX) [file pone.0177046.s003.docx]

**Table S3. Thr-138 related phosphopeptides identified from LC-MS/MS data of the tryptic digest of control and ROK-treated SNAP-25**

| Peptide | m/z | z | Control | ROK | Start | End |
| --- | --- | --- | --- | --- | --- | --- |
| RVT(Phospho)NDAR | 456.2084 | 2 |  | + | 136 | 142 |
| RVT(Phospho)NDARENEMDENLEQVSGIIGNLR | 1018.143 | 3 |  | + | 136 | 161 |
|  | 763.8593 | 4 |  | + |  |  |
| RVT(Phospho)NDARENEM(Oxidation)DENLEQVSGIIGNLR | 1023.475 | 3 | + | + | 136 | 161 |
|  | 767.8580 | 4 |  | + |  |  |
